# Distribution of Influenza-Like Illness (ILI) by Occupation in Washington State, September 2009–August 2010

**DOI:** 10.1371/journal.pone.0048806

**Published:** 2012-11-12

**Authors:** Naomi J. Anderson, David K. Bonauto, Z. Joyce Fan, June T. Spector

**Affiliations:** 1 Washington State Department of Labor & Industries, Safety & Health Assessment & Research for Prevention (SHARP) Program, Olympia, Washington, United States of America; 2 University of Washington, Environmental & Occupational Health Sciences Department, School of Public Health, Seattle, Washington, United States of America; Centers for Disease Control and Prevention, United States of America

## Abstract

**Objectives:**

*:* We aim to estimate the prevalence of influenza-like illness (ILI) by occupation and to identify occupations associated with increased ILI prevalence.

**Methods:**

Between September 2009 and August 2010, the Centers for Disease Control (CDC) included questions on ILI symptoms on the Behavioral Risk Factor Surveillance System (BRFSS). Washington State collects the occupation of all employed BRFSS respondents. ILI prevalence and prevalence ratios (PR) were calculated by occupational group.

**Results:**

There were 8,758 adult, currently employed, non-military respondents to the Washington BRFSS during the study period. The ILI prevalence for all employed respondents was 6.8% (95% Confidence Interval (95% CI) = 6.1, 7.6). PRs indicated a lower prevalence of ILI in Technicians (PR = 0.4, 95% CI = 0.2, 0.9) and Truck Drivers (PR = 0.2, 95% CI = 0.1, 0.7) and higher prevalence in Janitors and Cleaners (PR = 2.5, 95% CI = 1.3, 4.7) and Secretaries (PR = 2.4, 95% CI = 1.1, 5.4).

**Conclusions:**

Some occupations appear to have higher prevalence of ILI than others. These occupational differences may be explained, in part, by differing levels of social contact with the public or contact with contaminated surfaces at work, or by other occupational factors such as stress or access to health care resources.

## Introduction

Seasonal influenza is costly and associated with missed work and decreased work productivity. Annual United States medical costs for seasonal influenza are estimated to be $10.4 billion [1]. The total annual economic burden, which includes lost earnings, is estimated at $87.1 billion [1]. The mean number of lost work days per seasonal flu case ranges from three to five days. This estimate does not include diminished work productivity associated with continued work while ill [2].

Pandemic influenza also has potentially profound effects on the workforce. Compared to seasonal influenza, pandemic influenza results in greater morbidity and mortality in working age populations [3], [4], [5]. During the 2009 H1N1 influenza pandemic, working adults (18–64 years of age) had an eight to twelve times greater risk of being hospitalized or dying from H1N1 influenza compared to seasonal influenza [5].

Given the substantial burden of influenza on the workforce, accurate data about its prevalence and highest prevalence occupations are needed to guide prevention efforts. Flu virus transmission usually requires the close proximity of an infected person to a healthy person or via fomites. Therefore, healthcare and other occupations involving frequent contact with infected individuals may be at increased risk for influenza [6], [7], [8], [9], [10]. Prior research on influenza in healthcare settings has been limited to confirmed and hospitalized cases [6], [7], which may be an underestimate. There is also sparse data on influenza in occupations outside of the healthcare industry [6], [7], [9]. The US Centers for Disease Control and Prevention (CDC) have traditionally relied upon mortality, laboratory, hospitalization, and outpatient health care provider visit data for flu surveillance [11]. However, since many individuals with influenza do not seek medical care, the CDC initiated community-based surveillance of self-reported ILI symptoms (respiratory illness with fever, and cough or sore throat) through the Behavioral Risk Factor Surveillance System (BRFSS). Community-based surveillance of self-reported ILI on BRFSS began in September 2009 to assess the impact of the H1N1 Influenza pandemic [12]. This study estimated the prevalence of ILI by occupation and identifies occupations with higher prevalence of ILI using Washington State BRFSS data.

## Methods

### BRFSS Survey Questions

The BRFSS is an annual, CDC-funded, state-based, random digit-dialed, landline telephone survey of the non-institutionalized adult (≥18 years old) U.S. civilian population. BRFSS collects data on health-related behaviors and health conditions. In addition to the CDC Core questions, states may also select additional CDC modules or develop their own questions to include on their state’s BRFSS. BRFSS core questions collect data on age, gender, race/ethnicity, smoking status, weight, height, and current employment status.

Influenza vaccination status was determined by affirmative response to the question “A flu shot is an influenza vaccine injected into your arm. During the past 12 months, have you had a flu shot?” Or “During the past 12 months, have you had a flu vaccine that was sprayed in your nose? The flu vaccine sprayed in the nose is also called FluMist”.

The 2009 and 2010 Washington State BRFSS cooperation rate was 70.1% and 48.2% respectively, and the response rate was 68.9% and 47.5% respectively [13].

#### ILI questions

From September 1, 2009 through August 30, 2010, CDC sponsored a BRFSS module on influenza-like illness (ILI). ILI cases were determined by affirmative responses to two questions: “During the past month, were you ill with a fever?”, and “Did you also have a cough and/or sore throat?” Additional ILI questions were included in the BRFSS module, but the number of affirmative responses and/or number of eligible respondents were too small for meaningful interpretation in Washington State [14].

#### Occupation question and coding

BRFSS respondents are asked if they are currently employed, self-employed, out of work for more than one year, out of work for less than a year, a homemaker, a student or retired. For respondents who are self-employed or currently employed, Washington State BRFSS collects the respondent’s occupation by including the following question: “What is your job title?” If no job title was given, the respondent was then asked “What kind of work do you do?”.

Occupation was collected as narrative text responses and recorded verbatim. Responses were autocoded using the Standardized Occupation and Industry Coding (SOIC) software [15], [16] developed by the National Institute for Occupational Safety and Health (NIOSH). The SOIC program codes match a 1990 Census Bureau occupation code to the narrative text [17]. There were 8,917 BRFSS respondents during the time period. The majority (68.7%) of the occupational responses were successfully coded using the SOIC software. The remaining 29.9% of responses were manually coded by NIOSH-trained coders, who categorized the responses into one 3-digit occupational classification based on the National Center for Health Statistics Instruction Manuals [18] and SOIC [16]. There were 127 respondents who were excluded from our analysis as noncodable; and an additional 38 were deleted after restricting by age (18–89 years). The study population was 8,752 respondents.

#### Reference group

To identify occupations with higher prevalence of ILI, prevalence ratios (PRs) were calculated for all 29 individual occupations with more than 70 respondents compared to a reference group. The reference group was an aggregation of all other employed respondents in occupations with less than 71 respondents. The selection of the individual occupations was blinded to ILI status.

The reference group was significantly different (p<0.001) from the aggregate 29 occupations (see Table 1). The reference group had a lower mean age (42) than that of the aggregate occupations (43). The reference group had a higher percentage of males than the aggregated occupations; more Hispanic respondents and less respondents in the Other Race category than the aggregated occupations. The reference group also had lower rates of vaccination than the aggregated occupations (overall; and in both males and females).

**Table 1:** Differences between the reference group and the aggregate 29 selected occupations.

				Demographics								ILI							
				%								% ILI, 95% CI							
				Age		Gender		Race				Age		Gender		Months		Vaccinated	
	n	% ILI (95% CI)	ILI Prevalence Ratio (95% CI)	18–49	50–89	Male	Female	White, Non-Hispanic	Hispanic	All Other Races	Vaccinated	18–49	50–89	Male	Female	Sept 2009–March 2010	April 2010–Aug 2010	Yes	No
All Currently Employed	8,752	6.8 (6.1, 7.6)	–	69.2	30.8	54.8	45.2	81.1	8.1	10.8	20.8	7.6 (6.6, 8.6)	5.1 (4.2, 5.9)	5.8 (4.8, 6.8)	8.0 (7.0, 9.1)	7.6 (6.5, 8.6)	6.0 (4.9, 7.1)	7.0 (5.1, 8.9)	7.9 (6.8, 8.9)
Aggregate of all 29 selected occupations	4,265	6.8 (5.8, 7.8)	1.0 (0.8, 1.2)	67.7	32.3	51.1	48.9	82.0	6.4	11.6	22.1	7.2 (5.8, 8.6)	6.0 (4.7, 7.2)	5.2 (3.9, 6.5)	8.4 (6.8, 10.0)	8.2 (6.7, 9.7)	5.1 (3.8, 6.4)	8.3 (5.3, 11.2)	7.7 (6.2, 9.2)
Reference group: All other occupations (with ≤70 respondents)	4,487	6.8 (5.7, 7.9)	1.0	70.7	29.3	58.8	41.2	80.2	9.7	10.1	19.6	8.0 (6.5, 9.4)	4.1 (3.1, 5.1)	6.3 (4.8, 7.8)	7.6 (6.1, 9.1)	6.9 (5.6, 8.2)	6.8 (5.0, 8.5)	5.8 (3.3, 8.2)	8.0 (6.4, 9.6)

Note. CI = confidence interval; all data were weighted to account for BRFSS sampling design.

a The number of respondents for the vaccination status question was 6,139.

### Analysis

Using the coded occupational responses and the BRFSS ILI questions, descriptive statistics, ILI prevalence by occupational groups [17], and prevalence ratios were estimated. The data were weighted to account for the BRFSS sampling design (weighting by CDC to age-,race-,sex-specific state population estimates and respondent’s probability of selection).

All analyses were performed using STATA software (STATA Version 8.0, Stata Corporation, College Station, TX, USA 2003). The Washington State BRFSS survey questions and protocols were approved by the Washington State Institutional Review Board.

## Results

During the study period, there were 8,752 Washington BRFSS respondents who were 18 to 89 years of age, currently employed for wages or self-employed in non-military occupations. Demographics and vaccination information by occupation are presented in Table 2. Fifty-two percent of responses were from the period September 2009 through March 2010, while 48% were from April through August 2010. The respondents were 55% male and 45% female. Sixty-nine percent were between the ages of 18 and 49, and 31% were age 50–89. The median age was 43 years old. Eighty-one percent of the respondents identified as non-Hispanic White, and 8% as Hispanic.

**Table 2:** Demographics and vaccination rate by occupation in the Washington State employed population, BRFSS data, September 2009 to August 2010.

		%							
		Age		Gender		Race			
1990 Census Occupation	n	18–49	50–89	Male	Female	White, Non-Hispanic	Hispanic	All Other Races	Vaccinated^a^
All Currently Employed	8,752	69.2	30.8	54.8	45.2	81.1	8.1	10.8	20.8
Managers and Administrators, n.e.c.	651	65.4	34.6	68	32	88.3	2.7	9	16
Elementary School Teachers	471	61.9	38.1	35.3	64.7	90	5.7	4.3	19.6
Registered Nurses	268	64	36	7.5	92.5	86	2.6	11.4	58.9
Nursing Aides, Orderlies and Attendants	245	66.6	33.4	24.3	75.7	62.6	12	25.4	27.4
Supervisors and Proprietors, Sales Occupations	185	73.2	26.8	60	40	83.8	3.5	12.7	19.7
Administrative Support Occupations	180	73.4	26.6	28.5	71.5	86.8	2.4	10.8	25.7
Technicians, n.e.c.	136	72	28	75.2	24.8	87.8	3.2	9	20.9
Sales Workers, Other Commodities	133	70.2	29.8	57.7	42.3	86.4	5.4	8.2	20.2
Farmers, except Horticultural	124	71.6	28.4	73.4	26.6	37	59.6	3.4	7.1
Janitors and Cleaners	123	57.8	42.2	63	37	62.6	25.8	11.6	19.1
Management Related Occupations, n.e.c	118	69.8	30.2	34.4	65.6	81.1	2	16.9	20.7
Truck Drivers	110	73.2	26.8	94.2	5.8	88.7	8.5	2.8	16.4
Managers, Medicine and Health	105	47.1	52.9	37.9	62.1	88.1	3.9	8	35
Engineers, n.e.c	97	64.3	35.7	89.2	10.8	81.7	2.8	15.5	14.9
Administrators, Education and Related Fields	96	61.5	38.5	52.4	47.6	84.5	1.6	13.9	16.2
Secretaries	95	54.7	45.3	2.6	97.4	88.4	5.7	5.9	37
Bookkeepers, Accounting, and Auditing Clerks	95	55.7	44.3	6.1	93.9	86.8	2.4	10.8	21.1
Accountants and Auditors	94	68.6	31.4	37.5	62.5	91.6	0	8.4	25.1
Financial Managers	93	58.1	41.9	51.3	48.7	92.4	1.4	6.2	15.9
Teachers, n.e.c	92	67.4	32.6	36.1	63.9	96.5	1.3	2.2	5.4
Investigators and Adjusters, except Insurance	91	73.6	26.4	28.7	71.3	76.7	7.3	16	37.1
Postsecondary School Teachers	89	58.8	41.2	62.3	37.7	78.4	1.8	19.8	20.6
Management Analysts	88	56.2	43.8	67.3	32.7	74.3	5.9	19.8	12.2
Cashiers	88	86.6	13.4	22.7	77.3	72.3	10.7	17	19.5
Computer Systems Analysts and Scientists	83	82.9	17.1	82.2	17.8	54.3	7.7	38	23.9
Secondary School Teachers	83	66.4	33.6	44.7	55.3	83	5.9	11.1	27.7
Construction Laborers	80	69.2	30.8	91.1	8.9	74.5	21.3	4.2	19.4
General Office Clerks	80	66.7	33.3	21.1	78.9	84.8	2.7	12.5	34.9
Computer Programmers	72	90.4	9.6	89.8	10.2	85.8	1.2	13	11.5
Reference group: All other occupations(with ≤70 respondents)	4,487	70.7	29.3	58.8	41.2	80.2	9.7	10.1	19.6

Note. n.e.c. = not elsewhere classified; all data were weighted to account for BRFSS sampling design.

a The n for the vaccination status question was 6,139.

The ILI prevalence for all employed respondents was 6.8% (95% CI = 6.1, 7.6) (Table 3). The reference group had an ILI prevalence of 6.8%, which was not statistically different than the ILI prevalence of all employed respondents or that of the 29 selected occupations in aggregate (Table 1).

**Table 3:** Prevalence of Influenza like illness (ILI) by occupation and select characteristics, Washington State employed population, BRFSS September 2009 to August 2010.

			ILI (%)							
			Age		Gender		Months		Vaccinated^a^	
1990 Census Occupation	% ILI (95% CI)	ILI PR (95% CI)	18–49	50–89	Male	Female	Sept 2009–March 2010	April 2010–Aug 2010	Yes	No
All Currently Employed	6.8 (6.1, 7.6)	–	7.6	5.1	5.8	8.0	7.6	6.0	7.0	7.9
Managers and Administrators, n.e.c.	6.5 (4.2, 8.8)	0.9 (0.6, 1.4)	7.2	5.1	5.9	7.6	8.1	4.9	7.9	7.4
Teachers, Elementary School	5.1 (2.8, 7.3)	0.7 (0.5, 1.2)	4.8	5.6	4.7	5.2	7.0	2.8	1.0	7.5
Registered Nurses	5.7 (1.6, 9.9)	0.8 (0.4, 1.8)	7.7	2.3	–	6.1	7.9	3.4	9.2	4.7
Nursing Aides, Orderlies and Attendants	10.1 (4.9, 15.4)	1.5 (0.9, 2.6)	11.1	8.1	–	8.5	12.0	8.2	–	9.9
Supervisors and Proprietors, Sales Occupations	5.9 (2.3, 9.5)	0.9 (0.5, 1.6)	6.6	4.0	4.4	8.2	8.3	3.2	–	5.3
Administrative Support Occupations	9.5 (2.7, 16.3)	1.4 (0.7, 2.9)	10.8	6.0	–	7.4	12.4	6.2	–	10.8
Technicians, n.e.c.	3.0 (0.6, 5.3)	**0.4 (0.2, 0.9)**	2.4	4.4	1.5	–	4.3	1.4	–	4.7
Sales Workers, Other Commodities	4.0 (0.0, 8.0)	0.6 (0.2, 1.7)	4.1	3.5	1.2	7.6	5.3	2.2	–	5.3
Farmers, except Horticultural	4.0 (0.0, 9.2)	0.6 (0.2, 2.2)	–	1.2	5.4	–	2.1	–	–	2.1
Janitors and Cleaners	17.1 (6.5, 27.7)	**2.5 (1.3, 4.7)**	–	13.3	14.1	21.4	17.5	16.4	–	17.9
Management Related Occupations, n.e.c	6.1 (0.8, 11.5)	0.9 (0.4, 2.2)	7.0	4.1	–	8.6	10.9	2.0	–	1.0
Truck Drivers	1.6 (0.0, 3.4)	**0.2 (0.1, 0.7)**	1.5	1.8	1.7	–	1.0	–	–	2.9
Managers, Medicine and Health	7.9 (2.4, 13.4)	1.1 (0.6, 2.3)	–	8.6	–	10.5	8.9	–	–	–
Engineers, n.e.c	7.4 (0.0, 14.9)	1.1 (0.4, 3.0)	–	–	8.1	–	10.4	–	–	11.0
Administrators, Education and Related Fields	4.2 (0.3, 8.2)	0.6 (0.2, 1.6)	–	7.5	5.5	–	2.4	–	–	4.1
Secretaries	16.6 (3.7, 29.5)	**2.4 (1.1, 5.4)**	–	8.5	–	16.5	–	–	–	–
Bookkeepers, Accounting, and Auditing Clerks	9.4 (2.0, 16.8)	1.4 (0.6, 3.1)	–	6.7	–	9.8	12.6	–	–	13.1
Accountants and Auditors	5.4 (0.0, 11.6)	0.8 (0.2, 2.5)	–	–	–	8.3	5.3	–	–	14.9
Financial Managers	13.0 (3.1, 23.0)	1.9 (0.9, 4.2)	–	–	–	13.8	13.2	–	–	9.8
Teachers, n.e.c	4.2 (0.0, 8.7)	0.6 (0.2, 1.8)	–	–	–	6.6	3.4	–	–	6.3
Investigators and Adjusters, except Insurance	10.4 (2.5, 18.3)	1.5 (0.7, 3.3)	–	–	–	11.6	–	–	–	11.9
Postsecondary Teachers	6.2 (0.2, 12.2)	0.9 (0.3, 2.4)	–	7.1	–	–	6.4	–	–	–
Management Analysts	4.5 (0.0, 10.6)	0.7 (0.2, 2.6)	–	2.3	–	–	–	–	–	3.1
Cashiers	12.0 (2.2, 21.7)	1.8 (0.8, 4.0)	10.5	–	–	14.3	–	–	–	–
Computer Systems Analysts and Scientists	6.3 (0.0, 12.8)	0.9 (0.3, 2.6)	5.9	–	6.9	–	–	–	–	–
Secondary School Teachers	7.7 (0.0, 15.7)	1.1 (0.4, 3.2)	–	–	–	11.7	–	–	–	–
Construction Laborers	2.4 (0.0, 5.3)	0.3 (0.1, 1.2)	–	–	2.7	–	–	–	–	2.7
General Office Clerks	6.5 (0.6, 12.4)	0.9 (0.4, 2.4)	15.4	–	–	8.2	–	–	–	–
Computer Programmers	4.7 (0.0, 10.5)	0.7 (0.2, 2.4)	3.9	–	4.4	–	–	–	–	5.9
Reference group: All other occupations (with ≤70 respondents)	6.8 (5.7, 7.9)	1.0	8.0	4.1	6.3	7.6	6.9	6.8	5.8	8.0

Note. CI = confidence interval; n.e.c. = not elsewhere classified; all data were weighted to account for BRFSS sampling design; results for categories with <50 respondents are not reported.

a The number of respondents for the vaccination status question was 6,139.

The ILI prevalence for employed 18–49 year old’s was significantly higher than ILI prevalence in employed respondents aged 50–89 years, 7.6% vs. 5.1% (p<0.001) (Table 3). Women had a significantly higher prevalence of ILI, 8.0%, than men, 5.8% (p<0.001) (Table 3).

Respondents who identified themselves as non-Hispanic Whites had an ILI prevalence of 6.5%; respondents identifying as Hispanics had 8.1% ILI prevalence; and workers identifying as All Other Races had an ILI prevalence of 9.0% (Table 3). The difference in PR between non-Hispanic Whites and Hispanics was not significant; however, those of All Other Races had a significant PR of 1.4 (95% CI = 1.0, 1.9) indicating a slightly higher prevalence of ILI when compared to non-Hispanic Whites.

The highest prevalence of ILI was in female Janitors and Cleaners at 21.4% (Table 3), and Janitors and Cleaners consistently had weighted ILI prevalence across other characteristics of 13% or higher (Table 3), the highest of all the occupations examined.

The lowest weighted prevalence of ILI was found in Truck Drivers (1.6%) regardless of age or sex (Table 3). Technicians, n.e.c. (Technicians not elsewhere classified – in the Census 1990 titles excludes Health Care and Science Technologists and Technicians, as well as Engineering, Drafting, and Survey Technicians, however it may contain these workers if they reported only “Technician” in their narrative response) also had consistently low prevalence (Table 3).

Of the 6,139 respondents answering the influenza vaccination questions, 20.8% reported having received an influenza vaccination either by injection or nasal spray (Table 2).

Table 4 presents the PRs of nine select occupations (compared to the reference group) that could be of interest due to their hypothesized high levels of workplace interaction, such as Registered Nurses, Nursing Aides, Orderlies and Attendants, and Teachers (Elementary and Secondary School); as well as those found with notable high or low prevalence. Our ability to do stratified analyses was limited by the small sample size and the low prevalence of ILI. For these select occupations, PRs were calculated by age, gender, vaccination status, and by the presence or absence of children in the household (Table 4).

**Table 4:** Influenza like illness (ILI) prevalence ratios by select occupation and characteristics, Washington State employed population, BRFSS September 2009 to August 2010.

		PR (95% CI)										
			Gender		Age		Vaccinated^a^		Months		Children in Household	
Census 1990 Occupation	n	All	Males	Females	18–49 years	50–89 years	Vaccinated^a^	Unvaccinated	Sept 2009–March 2010	April 2010–Aug 2010	Yes	No
All occupations with >70 respondents	4,265	1.0 (0.8, 1.2)	0.8 (0.6, 1.2)	1.1 (0.8, 1.5)	0.9 (0.7, 1.2)	**1.5 (1.1, 2.0)**	1.4 (0.8, 2.5)	1.0 (0.7, 1.3)	1.2 (0.9, 1.6)	0.8 (0.5, 1.1)	0.9 (0.7, 1.3)	1.1 (0.8, 1.6)
Registered Nurses	268	0.8 (0.4, 1.8)	–	0.8 (0.4, 1.7)	1.0 (0.4, 2.2)	0.6 (0.2, 1.6)	1.6 (0.6, 4.1)	0.6 (0.1, 2.9)	1.1 (0.5, 2.7)	0.5 (0.1, 2.2)	1.0 (0.4, 2.3)	0.5 (0.2, 1.9)
Nursing Aides, Orderlies and Attendants	245	1.5 (0.9, 2.6)	2.4 (0.9, 6.9)	1.1 (0.6, 2.0)	1.4 (0.7, 2.7)	2.0 (0.8, 4.8)	0.8 (0.2, 3.3)	1.2 (0.6, 2.5)	1.7 (0.9, 3.2)	1.2 (0.4, 3.3)	0.8 (0.4, 1.7)	**2.6 (1.3, 5.2)**
Elementary School Teachers	471	0.7 (0.5, 1.2)	–	0.7 (0.4, 1.2)	0.6 (0.3, 1.2)	1.3 (0.7, 2.5)	0.2 (0.0, 1.2)	0.9 (0.5, 1.6)	1.0 (0.6, 1.8)	0.4 (0.2, 1.0)	0.6 (0.3, 1.2)	1.0 (0.5, 1.9)
Secondary School Teachers	83	1.1 (0.4, 3.2)	0.4 (0.1, 3.0)	1.5 (0.5, 4.9)	1.4 (0.5, 4.1)	**0.1 (0.0, 0.8)**	3.4 (0.6, 18.6)	0.7 (0.2, 2.8)	1.3 (0.4, 3.8)	0.9 (0.2, 6.5)	1.5 (0.5, 4.3)	**0.1 (0.0, 0.5)**
Technicians, n.e.c.	136	**0.4 (0.2, 0.9)**	**0.2 (0.1, 0.9)**	1.0 (0.4, 2.8)	0.3 (0.1, 1.0)	1.1 (0.4, 2.8)	0.5 (0.1, 3.8)	0.6 (0.2, 1.6)	0.6 (0.2, 1.6)	**0.2 (0.0, 0.8)**	**0.1 (0.0, 0.8)**	0.8 (0.3, 2.0)
Cashiers	88	1.8 (0.8, 4.0)	0.7 (0.1, 5.0)	1.9 (0.8, 4.5)	1.3 (0.5, 3.7)	**5.2 (1.9, 13.7)**	**4.4 (1.3, 14.5)**	1.9 (0.5, 6.3)	2.5 (0.9, 6.7)	0.9 (0.3, 2.8)	1.7 (0.5, 5.8)	1.8 (0.7, 4.7)
Secretaries	95	**2.4 (1.1, 5.4)**	4.3 (0.6, 33.1)	2.2 (1.0, 4.9)	**2.9 (1.1, 7.6)**	2.1 (0.8, 5.5)	**4.7 (1.0, 19.9)**	**3.3 (1.5, 7.2)**	2.3 (0.9, 5.7)	2.5 (0.8, 7.7)	2.8 (0.9, 8.5)	2.4 (0.8, 6.9)
Janitors and Cleaners	123	**2.5 (1.3, 4.7)**	2.3 (0.9, 5.4)	**2.8 (1.1, 6.9)**	**2.6 (1.1, 5.9)**	**3.2 (1.2, 8.5)**	–	**2.2 (1.0, 4.9)**	**2.5 (1.1, 5.9)**	2.4 (0.9, 6.4)	2.1 (0.9, 5.0)	**3.2 (1.3, 7.9)**
Truck Drivers	110	**0.2 (0.1, 0.7)**	**0.3 (0.1, 0.9)**	–	**0.2 (0.0, 0.8)**	0.4 (0.1, 2.1)	–	0.4 (0.1, 1.1)	**0.1 (0.0, 0.6)**	0.4 (0.1, 1.6)	**0.1 (0.0, 0.8)**	0.4 (0.1, 1.5)

Note. CI = confidence interval; PR = prevalence ratio; n.e.c. = not elsewhere classified; bold font indicates significance at p<0.05; all data were weighted to account for BRFSS sampling design; reference group for the ‘all occupations’ column, and for each row, is composed of those in that category in all occupations with ≤70 respondents.

a The original n for the vaccination status question was 6,139.

Analysis of the overall PRs shows significantly lower prevalence of ILI in both Technicians, n.e.c., PR = 0.4 (95% CI = 0.2, 0.9) and Truck Drivers, PR = 0.2 (95% CI = 0.1, 0.7); and significantly higher prevalence in Janitors and Cleaners, PR = 2.5 (95% CI = 1.3, 4.7) and Secretaries, PR = 2.4 (95% CI = 1.1, 5.4) when compared to reference group of occupations (Table 4). Secretaries, Janitors and Cleaners, and Cashiers all showed significant increased prevalence in some strata, and in some cases substantially (eg, Cashiers 50–89 years of age had a PR of 5.2 (95% CI = 1.9, 13.7), a strongly increased prevalence). Janitors and Cleaners also had consistently higher prevalence of ILI compared to the reference group, especially in older workers and those without children.

Registered Nurses did not have an increased risk of ILI compared with the reference group. Nursing Aides, Orderlies and Attendants showed consistently higher prevalence of ILI than Registered Nurses, and higher ILI prevalence than overall ILI prevalence and that of the reference group (Table 3), but analysis of the PRs found this elevation was only significant for those without children in the household (Table 4). Teachers (Elementary and Secondary School) also had a lower prevalence of ILI.

## Discussion

Analysis of the Washington BRFSS ILI data shows that the prevalence of ILI varies by occupation. Significantly higher prevalence of ILI was found in Janitors and Cleaners, and Secretaries; while there was a lower prevalence of ILI in Technicians, n.e.c., and Truck Drivers. To our knowledge, Janitors and Cleaners and Secretaries are occupations that have not previously been identified as having higher ILI prevalence. Other occupations that have been previously considered as having the potential for higher prevalence of ILI, such as Registered Nurses and Teachers (both Elementary and Secondary School), were not found to have any higher prevalence when compared to the reference group. In general, the distribution of ILI by age and gender in Washington BRFSS employed respondents was similar to US patterns for patients hospitalized with H1N1, in which the majority of patients were under 50 years of age [19]. Washington BRFSS ILI data showed a prevalence of 6.8%, which is similar to a national estimate of 8.1% [12]; however, because the WA BRFSS data is restricted to those currently employed and the national estimate is not, there are some demographic differences between populations.

### Gender

Previous research suggests a higher percentage of ILI in women (9.0%) compared to men (7.1%) and our data confirmed this observation [12].

The results do reflect a higher prevalence of ILI in women and healthcare workers are nearly 80% women [20]. However, when looking at ILI stratified by sex, neither Registered Nurses nor Nursing Aides, Orderlies and Attendants showed a higher prevalence of ILI. Low prevalence of ILI for Registered Nurses (and to a lesser extent Nursing Aides, Orderlies, and Attendants) may be at least partly explained by higher vaccination rates.

### Vaccination

The vaccination rate of the Washington employed population in our data was 20.7% (Table 2). These rates varied by occupation, with Registered Nurses having the highest vaccination rate, 58%, of the 29 selected occupations. There was a significant difference (p<0.001) in the vaccination rate between men (17.5%) and women (25%). Meta-analysis of predictors of seasonal influenza vaccination in health care workers (HCW) described male gender, age of 40 or older, and being a physician, as associated with a slightly increased chance of being vaccinated [21]. Knowing that the vaccine is effective, being willing to prevent influenza trasnmission, and believing that influenza is highly contagious, were among several beliefs on influenza that were identified as strong predictors of HCW being vaccinated [21]. These attitudes and beliefs may vary by education and occupation; there may also be differences in vaccination during pandemic influenza outbreaks as compared to seasonal influenza. Differences in vaccination rate by gender and occupation may account for some of the differences seen in ILI in the Washington State employed population as a whole, as well as between occupational groups in the Health care occupations.

### Occupation and Social Contact

Health care occupations (such as Registered Nurses and Nursing Aides, Orderlies, and Attendants) and teaching occupations have potentially high levels of contact with groups such as ill persons or children who may have a higher prevalence of ILI. HCW in particular are often targeted for prevention; and school closures have been identified as a way of social distancing to prevent the transmission of pandemic influenza [22]. However, both groups showed lower prevalence of self-reported ILI when compared to the other occupations analyzed.

In social contact modelling schools are considered a major source of influenza transmission [22], [23], [24], [25]. However, the studies focus primarily on transmission between students, and from students to their household contacts, not necessarily from students to teachers. One possible reason for the lower ILI prevalence found in teachers might be the level of contact with students. In one description of a pandemic flu outbreak in a London school, the only staff members who became ill were those who had cared for ill students – no other staff members became infected – presumably because they did not have such close contact with the infected [25]. In another UK outbreak involving teenage students in a summer program, those staying in university accomodations had the highest attack rate, with “no association found between illness and classes in specific teaching groups or classrooms” [24]. A description of an outbreak in a New York City high school identified self-reported ILI through an online questionnaire in 35% of student respondents but only in 10% of employee respondents [26]. A modelling study of social contacts and infectious disease [23] did identify young people (5–19 years old) as being expected to have a high incidence of a simulated emerging infection – but their contacts were highly assortative (within their own age and grade); overall, the authors concluded that contact “at home, school, or leisure were more likely to be physical than contacts at the workplace or while travelling” and “more-intimate contacts are likely to carry a greater risk of transmission… the most-intimate contacts occur at home or in leisure settings…” [23]. Teachers may indeed be around a population of young people with expected high incidence of ILI or other infections, but not in “close” or “intimate” contact with their students.

Socially isolative professions such as Farming, and Truck Driving had a lower prevalence of ILI than other occupations. Though we cannot estimate what kind of exposure Technicians, n.e.c. have to higher ILI prevalence populations, it could be that because they are involved in working with tools, machines, or other such technical work, that they have less close social contact with other persons at work or work more independently.

The occupations with the highest prevalence of ILI were Secretaries, and Janitors and Cleaners. Secretaries may have higher social contact with an infected person in a work setting. Shared office space has been shown to increase the risk for common colds [27], which is consistent with our observation that Secretaries have an increased prevalence of ILI. Janitors and Cleaners may have increased contact with contaminated surfaces, and may face a higher prevalence of ILI due to the close-contact and hands-on nature of their work, and high exposure to contaminated surfaces and materials (fomite transmission).

### Other Factors

Differences in PPE use, education, and training in infection control practices may partly explain the increased prevalence of ILI in Janitors and Cleaners. There may also be underlying demographic, socioeconomic, and employment-related factors contributing to differences in ILI prevalence [7], including job stress [28], [29], [30], [31], [32], job insecurity [33], availability of paid leave/benefits [34], and effort-reward imbalance (ERI) (which causes psychological distress that can negatively affect mental and physical health) [35].

Psychological stress can alter susceptibility to infections [28], [29], [30], is a risk factor for upper respiratory infections (URI) [28], [30], and can impair immune response to vaccinations [29]. One study found that individuals classified as “high stress” experienced signficantly more episodes of URI and more days with URI symptoms than individuals classifed as low stress or intermediate [30]. A study of stress and adherence to preventive measure for influenza in university students found that while higher levels of percieved stress did not affect facemask or hand hygiene compliance, higher levels of stress were significantly associated with a 25% increase in ILI incidence [31]. Percieved stress has also been associated with higher ILI reporting [32]. Job insecurity has also been associated with common infections and health complaints, with a 39% increased risk of self-reported ILI [33].

A study of sickness absence and work factors found sickness absence distributed on an occupational class gradient, with (male) manual workers and clerks having higher relative ratios of abscences for respiratory illnesses (4.21, 3.61 respectively) than engineers (1.14) and managers (reference group, 1.0), and the pattern was similar in women [36]; sickness absences for all causes was found to be attributable in part to work conditions such as ergonomic constraints and work stress in both men and women [36].

A lack of access to paid leave may also negatively affect workers in certain occupations. An evaluation of the impact of workplace policies fond that the absence of policies such as paid sick leave contributed a population-attributable risk of 5 million additional cases of ILI, and in particular, 1.2 million of those among Hispanics (also found in the same study to have higher percieved job insecurity) [34]. Of the WA BRFSS respondents, Janitors and Cleaners had the second highest proportion of Hispanic workers (25.8%, Table 3), behind farm workers. Analysis of BLS Employee Benefits surveys found that sick leave coverage among occupations varied considerably – of those in service occupations, only 37% had access to paid sick leave, and some occupational groups had even less, specifically handlers, equipment cleaners, helpers, and laborer occupations (35%) and machine operators, assemblers and inspectors (29%) [37]. In comparison, 73% of executive, administrative and managerial occupation workers had paid sick leave coverage, professional/technical occupations 71%, and administrative support and clerk occupations, 68% [37]. Access to paid sick leave was also found to be “largely restricted to workers in the top three wage quartiles… within almost every industry…” and this lack of access was noted to be particulary burdensome to women, who make up 60% of minimum-wage workers [37].

Of WA BRFSS respondents, female janitors and cleaners had the highest prevalence of ILI (21.4%, Table 3). A study of Las Vegas hotel room cleaners found that hotel room cleaners (99% female, 84% immigrant) had a high prevalence of effort-reward imbalance (ERI), which was significantly associated with the population’s high prevalence of poor or fair (self-rated) general health [35].

### Health Care Workers

Compared with other occupations, certain health care professionals (Registered Nurses, and Nursing Aides, Assistants and Orderlies) did not have a higher prevalence of ILI. These results are consistent with a previous study [8], where HCW in four hospitals did not face an increased risk for H1N1 influenza during the 2009 pandemic when compared to a non-clinical control population. Compliance with use of personal protective equipment in HCW such as PPE, attention to hand hygience, and other infection control precautions were suggested as an explantion for this observation. Lack of compliance with PPE and infection control precautions were also noted to possibly play a role in transmission of pandemic influenza to HCW [38]. Increased attentiveness to infection control precautions may also explain some differences in HCW prevalence of pandemic influenza outbreaks when compared to seasonal influenza.

Other studies have shown an increased prevalence of seasonal influenza for all health care workers (HCW) [9]. In a post-hoc analysis, we compared prevalence of ILI among different health care occupations. There was a difference in ILI prevalence between Health Diagnosing and Treatment occupations (Physicians, Registered Nurses, Health Care Managers, and Dentists) and that of Health Technologists, Health Technicians, Nursing Aides, Orderlies, and Attendants. Workers in the Health Diagnosing and Treatment occupations group had an ILI prevalence of 4.9%, whereas workers in the other HCW occupations group had an ILI prevalence of 8.9% (p<0.001). However, the PR for the comparison between Health Diagnosis and Treatment occupations such as Physicians and Registered Nurses (used as the reference group), and that of Nursing Aides, Orderlies and Attendants was not significant (PR = 1.7; 95% CI = 0.9, 3.2, p<0.08).

Given the differences in ILI prevalence by race and gender seen in our small sample, future work should further explore the potential reasons for these differences. Occupational segregation by race or gender might partially explain the higher prevalence of ILI in non-white and Hispanic populations, although our sample was too small to test this. Future work could also benefit from some measure of the contact rates in social and occupational networks; O*NET [39] provides a ranking of occupations by “Physical Proximity”, which assigns a score according to “what extent does this job require the worker to perform job tasks in close physical proximity to other people.” However, the occupations reported by the WA BRFSS respondents generally had higher scores of contact (when crosswalked to the O*NET titles; data not shown) and the ranking was of limited value.

### Limitations

While there are some differences in the distribution of the population between the reference group (those occupations with ≤70 respondents) and the distribution of all of the employed, there was no difference in ILI PR overall between the reference group and our aggregate 29 presented occupations (Table 1).

Multivariate modeling by occupation would be preferable but would have required a larger surveyed population than what we had available. However, the observed results seem biologically plausible in terms of higher or lower exposure (direct social contact or via fomites) resulting in higher or lower risk of ILI by occupation when vaccination rates are considered.

Some possible bias towards the null may have been introduced by assignment of nonspecific narrative responses to larger occupational groups, such as those occupations not elsewhere -classified (n.e.c.).

Another important limitation is that the ILI questions measure non-specific symptoms attributable to many other illnesses like the common cold. Teachers and other school workers have been found to have a higher prevalence of head and chest colds during the school year than all other workers [40]. A study using clinical case definitions of flu-like illness, as fever with cough, deemed it “imperfect” and found a positive predicitive value of 86.8%, sensitivity of 77%, and considered it helpful only when influenza is known to be circulating [41]. Individuals may also percieve and self-report their illnesses differently, and this might also vary by occupation (some HCW for example, who might be more knowledgeable about ILI, may be more or less likely to self-report with ILI). However, because of the widespread nature of the flu, and that most people do not seek treatment, using such a definition of ILI in a telephone survey is an important surveillance tool to better estimate otherwise unrecognized ILI.

Since characterizing the degree of social contact necessary to complete work is difficult, OSHA guidance to prepare workplaces for an influenza pandemic recommends limiting “close contact (within 6 feet)” whenever possible, even for occupations at lower and medium exposure risk [42].

### Strengths

We are not aware of any other published study analyzing self-reported ILI prevalence by occupation. This analysis provides valuable insight into the occurrence of ILI in a working population. Additionally, our data are representative of employees in Washington State, with the weighted estimates of the employed Washington population in BRFSS being consistent with estimates of the Washington employed population from other data sources [43].

BRFSS is a national-scale validated survey system that has been used for many years to collect health and behavioral information and set policy. The use of occupational data in this manner is a unique approach to differentiate ILI prevalence within an employed population, and could extend to assisting in pandemic flu planning. If industry and occupation data are collected in more states, or nationally, more occupations could be analyzed and the results would have improved power and the ability to explore differences by race and gender. Even at this limited level, however, one can differentiate ILI prevalence by occupation in the employed Washington population, demonstrating that further research using occupation data to characterize ILI is warranted.

### Conclusions

Some occupations appear to have higher prevalence of ILI than others. Some of these differences may be explained, at least in part, by differing levels of social contact and other factors such as stress and access to leave by occupation. Given the serious impact of the flu on the working population, targeting of prevention resources should take occupational exposures into account. Routine collection of industry and occupation information in BRFSS and other surveillance instruments would help in identifying industries and occupations with higher prevalence of ILI or other outcomes of interest and tailoring prevention strategies accordingly.
